# β-glucans and eicosapolyenoic acids as MAMPs in plant–oomycete interactions: past and present

**DOI:** 10.3389/fpls.2014.00797

**Published:** 2015-01-13

**Authors:** Sara M. Robinson, Richard M. Bostock

**Affiliations:** Department of Plant Pathology, University of CaliforniaDavis, Davis, CA, USA

**Keywords:** arachidonic acid, eicosapolyenoic acid, innate immunity, oligosaccharins, oxylipins, *Phytophthora*

## Abstract

Branched β-1,3-glucans and the eicosapolyenoic acids (EP) are among the best characterized oomycete elicitors that trigger innate immune responses in plants. These elicitors were identified over three decades ago, and they were useful in the study of the sequence of physiological, biochemical and molecular events that induce resistance in plants. However, in spite of the cross-kingdom parallels where these molecules are well-characterized as immune system modulators in animals, their perception and modes of action in plants remains obscure. Oomycetes are among the most important plant pathogens, responsible for diseases that devastate crops, ornamentals, and tree species worldwide. With the recent interest and advances in our understanding of innate immunity in plants, and the redefining of many of the classical elicitors as microbe-associated molecular patterns (MAMPs), it seems timely and important to reexamine β-glucans and EP using contemporary approaches. In this review, we highlight early studies of β-glucans and EP, discuss their roles as evolutionarily conserved signals, and consider their action in relation to current models of MAMP-triggered immunity.

## INTRODUCTION

Over 30 years ago branched β-1→3-glucans and the EP – AA and EPA – were characterized as potent oomycete elicitors of innate immune responses in plants. These and the *Phytophthora* elicitin proteins with activities in a somewhat narrower host range (Tyler, 2002) figured prominently in the literature in subsequent years, and were used to examine physiological, biochemical and molecular events associated with the HR and induced resistance. Intriguing is that β-glucans and EP are important in modulating innate immunity and inflammation in animals, although these cross-kingdom parallels are likely not fully appreciated by the plant and animal research communities.

Oomycetes are among the most important plant pathogens, responsible for devastating plant diseases worldwide. New *Phytophthora* species, in particular, are continually being discovered, with the number of species identified nearly double that of only a decade ago (Hansen et al., 2012; Kroon et al., 2012). Downy mildew pathogens and the diseases they cause are also current threats to U.S. and world agriculture, with two listed as Select Agents as serious threats to U.S. agriculture (http://www.selectagents.gov). The *Phytophthora* research community is attuned to the need and urgency to develop novel control strategies that are broadly applicable yet sustainable, with vigorous research programs studying population genetics, genomics, effector biology, host resistance, and disease epidemiology and management. Within this research portfolio, determining how β-glucans and EP are perceived and act in plants could be useful for enhancing disease resistance against oomycetes and possibly other attackers. In this review, we highlight early studies of β-1→3-glucans and EP, discuss their roles as evolutionarily conserved signals, and consider their action in relation to current models of MAMP ^1^-triggered immunity.

## EICOSAPOLYENOIC ACIDS

**FIGURE 1:**
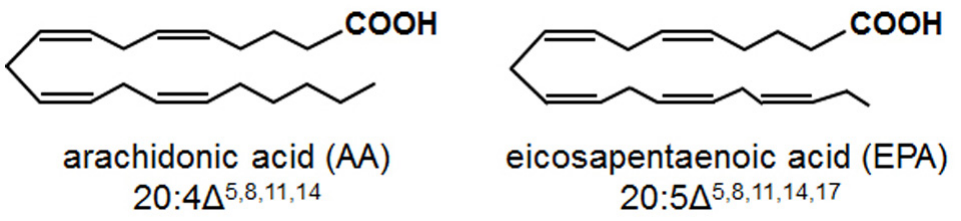
**Chemical structures of the eicosapolyenoic acids, arachidonic acid, and eicosapentaenoic acid**.

Arachidonic acid (AA; 20:4 Δ^5,8,11,14^) and eicosapentaenoic acid (EPA; 20:5 Δ^5,8,11,14,17^) are 20-carbon, all-*cis* PUFAs containing four and five double bonds, respectively (**Figure 1**). In mammals, AA and EPA undergo enzymatic oxidation to oxylipins, referred to as eicosanoids, which serve crucial signaling functions in stress responses (Blee, 2002; Bostock et al., 2011). Examples of these eicosanoids include prostaglandins and thromboxanes, formed via the action of cyclooxygenases, and leukotrienes, formed via the action of LOXs. Eicosanoid-mediated stress responses include pain, inflammation and fever (prostaglandins), platelet aggregation and vasoconstriction (thromboxanes), and allergic responses and asthma (leukotrienes; De Caterina and Basta, 2001; Murakami, 2011). Although higher plants do not contain AA and EPA, AA and EPA are found in oomycete pathogens and plants are exposed to these fatty acids during infection (Walley et al., 2013).

Many molecules of microbial pathogens identified as elicitors in earlier studies have been reclassified as MAMPS to conform to terminology used in animal immunity. MAMPs are motifs in essential molecules such as proteins, lipids, and polysaccharides that are present in entire classes of microbes (pathogenic or non-pathogenic). These molecular motifs are generally absent from hosts and can be recognized by plants and animals, such as in response to attempted infection or colonization. Defense responses induced by MAMPS in plants are referred to as PTI (Nurnberger et al., 2004; Boller and Felix, 2009; Zipfel and Robatzek, 2010). Studies of PTI have focused on the bacterial peptides flagellin and EF-Tu and their action in *Arabidopsis*. These peptides are perceived by PRRs, receptor-like kinases that are crucial for perception of flagellin/EF-Tu and activation of PTI. However, unlike flagellin and EF-Tu, many of the historical elicitors that stimulate well-characterized defense responses in plants have not been sufficiently investigated to resolve their modes of action (Nurnberger et al., 2004; Boller and Felix, 2009; Nguyen et al., 2010; Zipfel and Robatzek, 2010). The elicitors AA and EPA conform to the definition of MAMPs: they are not present in higher plants, are essential components in oomycete cells, are largely absent from other classes of microbes, and elicit similar defense responses in plant species where they have been studied (Tyler, 2002; Bostock et al., 2011; Walley et al., 2013).

Eicosapolyenoic acid elicitor activity in plants was first discovered in the interaction between *Phytophthora infestans* and potato. Mycelial extracts of *P. infestans* induced sesquiterpenoid phytoalexins, lignin deposition and cell death in potato tissue in a reaction similar to a HR to incompatible races of the pathogen. Purification and analysis of all active fractions in these extracts identified AA and EPA, without exception, either free or esterified to other molecules (Bostock et al., 1981, 1982). Elicitation was specific to AA and EPA. Treatment with 15 other fatty acids, including LA (18:2^Δ9,12^) and ALA (18:3^Δ9,12,15^), the primary unsaturated fatty acids found in higher plants (Kachroo and Kachroo, 2009), as well as structurally similar eicosatrienoic acid (20:3^Δ11,14,17^) and arachidonyl alcohol, did not elicit defense responses. Treatment of tuber disks with AA also protected them from subsequent *P. infestans* infection (Bostock et al., 1981, 1982).

## EP-INDUCED RESISTANCE AGAINST PATHOGENS AND PHYTOHORMONE DEFENSE SIGNALING

Eicosapolyenoic acids induce systemic resistance in potato as well as in other plant species to various pathogens. Although the mechanisms remain unresolved, EP have been shown to elicit SA, JA, and ET in different experimental systems. Colonization of avocado seedling roots by *P. cinnamomi* was reduced in roots treated with AA prior to inoculation (Romero-Correa et al., 2014). Pearl millet seedlings were protected to a greater degree against infection by the downy mildew pathogen, *Sclerospora graminicola*, following seed treatment with AA or EPA, in contrast to seedlings emerging from seeds treated with LA, ALA, DHA or water (Amruthesh et al., 2005).

EP elicit SAR or SAR-like responses in tobacco, potato, and tomato. Treatment of lower leaves of tobacco plants with AA induced local and SAR to TMV (Rozhnova et al., 2003). EP treatment of the lower leaves of potato plants protected the upper leaves from infection by *P. infestans*, a systemic resistance that developed within 5 days of the inducing treatment (Cohen et al., 1991). Plants treated with LA, ALA, or oleic acid displayed partial protection but not to the level of EP-treated plants. AA also induced resistance in potato leaves to the early blight pathogen, *Alternaria solani*, with levels of SA and a PR1-like protein elevated in the AA-treated leaves (Coquoz et al., 1995). AA-treatment of tomato leaves induced localized accumulation of transcripts for *P4* (Fidantsef et al., 1999), a PR-1 family member and SAR marker in tomato (Van Kan et al., 1992), but did not induce expression of the proteinase inhibitor gene *PI-2*. The latter is strongly induced by wounding and JA treatment and serves as a marker for JA-mediated resistance in tomato (Fidantsef and Bostock, 1998; Fidantsef et al., 1999).

Although the studies in tobacco, potato, and tomato indicate that EP-induced resistance may operate through SA, recent research suggests EP action is more complex (Savchenko et al., 2010). Treatment of tomato and *Arabidopsis* leaves with AA increased JA levels, reduced SA levels, and increased resistance to *Botrytis cinerea*. *Arabidopsis* plants transformed to produce small amounts of EPs (named EP plants) were less susceptible to *P. capsici*, *B. cinerea*, and feeding by aphids. However, these plants were more susceptible to *Pseudomonas syringae* pv. *tomato* (DC3000). The EP plants had constitutively elevated levels of JA and JA-marker gene expression and reduced levels of SA and SA-marker gene expression relative to wild-type plants. The differential effect of EP on disease and pest outcomes corresponds to EP’s impact on SA and JA defense signaling, and this effect is dependent upon JA as demonstrated with a JA-deficient *aos* mutant line (Savchenko et al., 2010).

Salicylic acid and JA can be mutually antagonistic (Bostock, 2005), making it difficult to reconcile these different findings. AA treatment elicits ET production in both pepper and potato (Bostock et al., 1986; Garcia-Pineda and Lozoya-Gloria, 1999), and ET can modulate SA- and JA-defense networks (Pieterse et al., 2012). The different experimental outcomes may result in part from differences in EP concentrations used in the various studies. Higher concentrations of EP can induce an intense, localized necrosis at the site of application, particularly in solanaceous plants. This strong phenotype could trigger or result from phytohormone changes different from those induced by low concentrations. Also, it is possible that all three phytohormones (SA, JA, and ET) are important in establishing EP-induced resistance through a process of transitional signaling (Truman et al., 2010). A study in potato indicates that both SA and JA are important in PTI responses (Halim et al., 2009), and a study of PTI in *Arabidopsis* using signal allocation analysis of mutants deficient in ET, SA, and JA signaling indicated that PTI depends on synergy among ET, SA, and JA (Tsuda et al., 2009). Further research is needed to fully elucidate the interactions among SA, JA, and ET in their involvement in EP-induced resistance and defense responses.

## PHYTOALEXIN INDUCTION

Eicosapolyenoic acids have been useful in dissecting aspects of secondary metabolism in plants, with a focus on sesquiterpenoid phytoalexins in solanaceous plants. However, EP elicits production of defense metabolites in other plant families as well. The isoflavanoid phytoalexins phaseollin and coumestrol accumulate in leaves of French bean following infiltration with AA (Longland et al., 1987). Phenol-2,4-bis (1,1-dimethylethyl), a defense compound in avocado, is induced in roots treated with AA as well as with SA (Romero-Correa et al., 2014). Among solanaceous plants EP elicit sesquiterpenoid phytoalexin synthesis in thorn-apple, eggplant, chili pepper, green pepper, potato, and tomato (Bloch et al., 1984; Whitehead et al., 1990; Hoshino et al., 1994; Castoria et al., 1995; Garcia-Pineda and Lozoya-Gloria, 1999). In potato tuber, AA elicits sesquiterpenoid phytoalexin biosynthesis with strong expression of sesquiterpene cyclase, a committed step in the pathway. Concurrent with this is a complete suppression of wound-induced squalene synthase and steroid glycoalkaloid accumulation (Tjamos and Kuć, 1982; Zook and Kuć, 1991). HMGR catalyzes the first step in the synthesis of stress-induced isoprenoids from mevalonate in potato. Three isoforms of HMGR are differentially induced by wounding and AA treatment (Choi et al., 1992), and a similar expression pattern of the corresponding HMGR isoforms occurs in tomato (Rodriguez-Concepcion and Gruissem, 1999).

## GENERATION OF REACTIVE OXYGEN SPECIES/PROGRAMMED CELL DEATH

In addition to potato, EP have been shown to elicit PCD, characteristic of the HR, in other plant species. Pearl millet seedlings treated with AA displayed a HR similar to that induced by the oomycete, *S. graminicola*, the causal agent of downy mildew. Following treatment with AA, the HR developed more quickly in pearl millet seedlings with genotypes rated as resistant versus susceptible to *S. graminicola* (ratings were based on field studies; Geetha et al., 1996). Tomato protoplasts treated with AA underwent PCD with characteristic DNA fragmentation and laddering, while LA and ALA treatment had no PCD-inducing effects (Knight et al., 2001).

In both potato and pepper, AA was found to induce ROS in a similar manner. AA treatment of potato tuber disks elicited a biphasic oxidative burst (generation of ROS) peaking at 1 and 6–9 h after treatment and increased expression of *StRBOHB*, a homolog of *gp91*(*phox*), which encodes a subunit within the neutrophil NADPH oxidase complex (Yoshioka et al., 2001). As in potato, treatment of pepper fruit with AA elicited an immediate, rapid ROS burst. When DPI, an inhibitor of NADPH-dependent oxidases, was applied to the fruit prior to application of AA, ROS generation decreased as the concentration of DPI was increased (Araceli et al., 2007).

## HOW DO AA AND EPA ELICIT DEFENSE RESPONSES?

The mode of action of EP in PTI is unresolved, although the structural requirements of EP as elicitors are well characterized. These include at least a 20 carbon backbone with all *cis*-1,4-pentadiene unsaturation beginning at the Δ5 position and at least four double bonds in the chain (Bostock et al., 1981, 1982; Preisig and Kuć, 1985; Savchenko et al., 2010). While this specificity could provide evidence for involvement of a receptor that recognizes these structural features, previous studies of EP in potato indicate that initial perception by plant cells may be quite different than other MAMPs. Initial recognition of AA and EPA may occur by specific disruption of host membrane integrity and/or perturbation of oxylipin metabolism, with the possibility that plant cells produce novel oxylipins from EP (Bostock et al., 1992; Ricker and Bostock, 1992, 1994; Fidantsef and Bostock, 1998). Studies in potato showed that U-^14^C radiolabeled AA was quickly incorporated into neutral lipids (mono-, di-, and tri-glycerides) and polar lipids (glycolipids and phospholipids). A small fraction, ∼2–5% of the AA, was oxidized (Preisig and Kuć, 1988; Ricker and Bostock, 1992). Also, sporangia of *P. infestans* readily incorporated exogenous ^14^C-AA into phospholipids (primarily), diglycerides and TGs. By 12–14 h after inoculation, microautoradiographic studies revealed that the radioactivity from sporangia was released into the epidermal and palisade mesophyll cells adaxial to the inoculated leaf surface and distant from fungal structures (Ricker and Bostock, 1992). Plant phospholipases are activated following attack by pathogens or treatment of plants with elicitors (Bostock, 1989; Canonne et al., 2011). This could create an opportunity for any EP incorporated into plant lipids during infection to be released and accessible to plant oxylipin enzymes.

**FIGURE 2:**
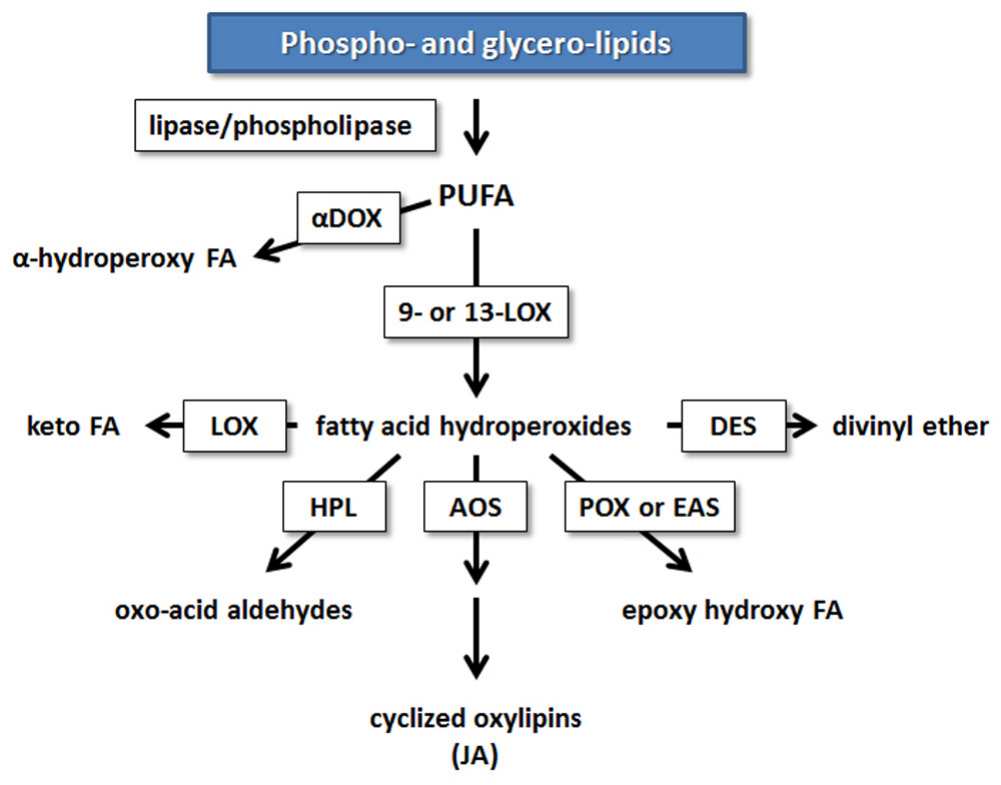
**Enzymatic mechanisms leading to the synthesis of oxylipins in plants from PUFA.** Enzymes involved in oxylipin metabolism are boxed. AOS, allene oxide synthase; αDOX, α-dioxygenase; DES, divinyl ether synthase; EAS, epoxy alcohol synthase; FA, fatty acid; HPL, hydroperoxide lyase; HPR, hydroperoxide reductase; JA, jasmonic acid; LOX, lipoxygenase; POX, peroxygenase [modified from (Shah, 2005)].

Research in potato and tomato indicates that the 9-LOX pathway may play an important role in EP action. The first step in the enzymatic formation of phyto-oxylipins involves the action of LOX (**Figure 2**). Plant LOXs act on PUFA containing a cis-(1,4)-pentadiene system, inserting an oxygen molecule (O_2_) to form hydroperoxy fatty acids. These are further metabolized to various oxylipin families by members of CYP74 cytochrome P450s: AOSs, HPLs, and DESs, or by less well-characterized POX or PXG and EASs (Blee, 2002; Feussner and Wasternack, 2002; La Camera et al., 2004; Kachroo and Kachroo, 2009; Mosblech et al., 2009).

**FIGURE 3:**
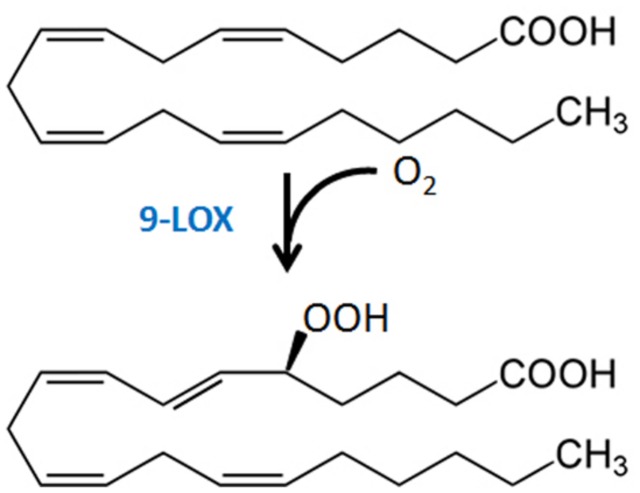
**Formation of 5-HPETE from AA via plant 9-LOX action**.

The importance of LOX, in particular a 9-LOX ^2^, in EP elicitor activity is supported by fatty acid structure-activity requirements and studies of LOX expression. The carboxyl function of EP is critical, a feature consistent with the substrate requirement of plant LOXs (Preisig and Kuć, 1985; Feussner and Wasternack, 2002). A Δ5 double bond at the beginning of a methylene-interrupted series with at least four double bonds provides the highest elicitor activity (Bostock et al., 1981, 1982; Preisig and Kuć, 1985; Feussner and Wasternack, 2002). AA stimulates LOX expression in potato and tomato (Bostock et al., 1992; Robinson et al., 2014), with 5-HPETE (**Figure 3**) a principal LOX product formed after treatment of tissue with AA (Ricker and Bostock, 1994; Robinson et al., 2014). Expression of *pLOX1*, a potato LOX gene now identified as a 9-LOX type 1 (Andreou et al., 2009), was strongly induced in AA-treated and *P. infestans*-inoculated potato tuber disks and leaves (Fidantsef and Bostock, 1998), as was a tomato LOX in AA-treated tomato leaves (Fidantsef et al., 1999). LA-treatment did not induce *pLOX1* expression or LOX activity. Heat treatment of tuber disks inactivates enzyme activity and abolishes HPETE formation following AA treatment (Ricker and Bostock, 1994), and EP-induced responses are strongly diminished when LOX activity is inhibited or absent (Preisig and Kuć, 1987; Vaughn and Lulai, 1992). Nonetheless, definitive experiments with LOX knock-out/knock-down or overexpression lines to critically test specific LOX isoforms in EP action have not been reported. While it has been proposed and is quite likely that the 9-oxylipin pathway metabolites of AA may directly act as signal molecules to activate defense responses (Regdel et al., 1994), AA and/or its metabolites may also induce expression and activity of oxylipin pathway enzymes to form biologically active metabolites from the plant LA and ALA pools.

Studies during the past 15 years in solanaceous plants point to the importance of 9-LOX and the 9-oxylipin pathway in defense, and have demonstrated that the 9-LOXs from potato, tobacco, and pepper can utilize AA as a substrate. Many of these studies have investigated defense responses against oomycete pathogens or used elicitor preparations from oomycetes likely containing EP (Fournier et al., 1993; Veronesi et al., 1996; Gobel et al., 2001, 2002; Andreou et al., 2009; Hwang and Hwang, 2010). 9-hydroperoxy fatty acids can be utilized by downstream oxylipin pathway enzymes to form other compounds that have been found to function in defense. In particular, DESs are induced in response to elicitors and pathogen attack in several solanaceous species including potato, tobacco, and pepper (Weber et al., 1999; Stumpe et al., 2001; Fammartino et al., 2007; Gullner et al., 2010). DESs are CYP74D P450s that produce the divinyl ethers CA from 9-HPOD and CnA from 9-HPOT.

Recent experiments indicate that treatment of tomato roots with EP induces resistance against *P. capsici*. Hydroponically grown tomato plants whose roots were treated with EP and subsequently inoculated with *P. capsici* experience significantly less rot and collapse at the crowns than plants whose roots were treated with H_2_O, LA, or ALA, indicating that exposure of tomato roots to EP prior to inoculation with *P. capsici* reduces susceptibility of the plants to *P. capsici* (Roberts et al., 2013). Further experiments demonstrate that roots and crowns display significantly increased lignification responses following root treatment with AA and EPA and subsequent inoculation with *P. capsici* compared to roots treated with H_2_O, LA, and ALA. AA-treatment of tomato roots elicits increased expression of 9-LOX and 9-DES genes in tomato roots compared to control treatments (LA and H_2_O). Expression of 9-DES is also increased following inoculation of roots with *P. capsici* (Robinson et al., 2014).

In conclusion, although EP action in plants is complicated, evidence supports an important role for LOX and likely a 9-oxylipin pathway in the initiation of plant responses. Furthermore, in *Arabidopsis* an intact JA pathway is required for AA activity, implicating a 13-LOX. Whether DES and divinyl ethers participate in the plant response to EP observed in solanaceous plants is unresolved, although ongoing research in our laboratory will address this issue. The search for a traditional PRR for EP in plant cells analogous to those for other MAMPs, although intriguing, may not be productive given other mechanisms for rapid uptake of PUFA by plant cells and their entry into oxylipin metabolism.

## β-GLUCANS AND RELATED OLIGOSACCHARINS IN PLANT IMMUNITY

β-linked glucose polysaccharides are the most abundant component of *Phytophthora* cell walls, comprising more than 80% of the wall dry weight (Bartnicki-Garcia and Wang, 1983). These include insoluble β-1→4-linked (cellulosic) and β-1→3, β-1→6-linked glucans, with the latter by far the more abundant of these polymers. In addition to the abundance of glucose, compositional analyses of cell walls also reveal minor amounts of mannose and glucosamine, as well as protein and lipid similar to levels found in cell walls of fungi. In addition to the insoluble glucans, soluble β-1→3-linked glucans are present at various developmental stages in the oomycete life cycle. For example these can be found in the germination fluids of cystospores as well as other stages, and during synthesis and remodeling of the wall during growth, thus making them potentially available at the host–pathogen interface during infection (Doke et al., 1980; Waldmuller et al., 1992). Laminarans are linear β-1→3-linked glucans that provide the dominant storage carbohydrate in *Phytophthora* and other oomycetes, as well as other stramenopiles (Bartnicki-Garcia and Wang, 1983).

**FIGURE 4:**
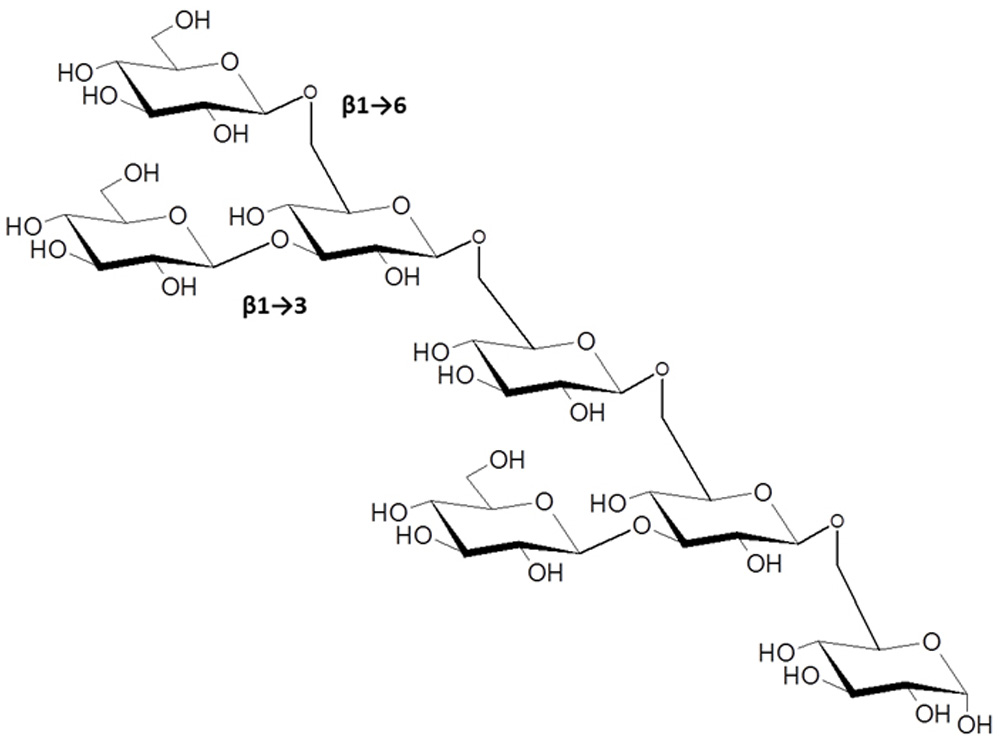
**The β-1→3, 1→6-linked hepta-β-glucoside from *Phytophthora sojae*, with potent elicitor activity in members of the Fabaceae (from Cheong et al., 1991).** Other β-1→3, 1→6-glucans with higher degrees of polymerization have immunomodulatory functions in plant-microbe interactions as discussed in the text.

The β-1→3, β-1→6-linked glucans present a very complex array of possible structures, some with well-established activity in modulating plant innate immunity. The most prominent example is the elicitor activity associated with glucans isolated from cultures and cell walls of the soybean pathogen *P. sojae* (formerly *P. megasperma* f. sp. *glycinea*). Albersheim et al. (1983) showed that these were potent inducers of the flavonoid phytoalexin, glyceollin, and related defense reactions in soybean cotyledons. β-glucan oligosaccharide fractions of varying complexity had elicitor activity suggesting a model whereby cell wall fragments released during infection provide the physiological triggers of the plant defense response. The smallest active fragment following partial acid hydrolysis of *P. sojae* cell walls was purified and shown to be a hexa (β-D-glucopyranosyl)-D-glucitol. This oligosaccharide and its corresponding unreduced hepta-β-glucoside elicited at concentrations between 10^-7^ to 10^-9^ M (Sharp et al., 1984; **Figure 4**). Subsequent work by Michael Hahn and coworkers further defined the branched β-1→3, β-1→6 structural motif essential to maximally induce phytoalexin accumulation (Cheong et al., 1991) and found that the hepta-β-glucoside specifically bound to soybean membranes with high affinity (Cheong and Hahn, 1991). These investigators provided strong evidence that the binding activity was associated with a membrane protein or glycoprotein. Subsequent efforts by other laboratories identified hydrophobic membrane proteins that bind β-glucans with high affinity from soybean (Cosio et al., 1992; Umemoto et al., 1997; Mithofer and Ebel, 1999) and other legumes (Mithofer et al., 1999). Reconstitution of the soybean homolog in lipid vesicles strongly bound the hepta-β-glucoside (*K*_d_ = 6–7 nM, with even higher affinities reported in other studies), which could be displaced by glucans with different degrees of polymerization in competitive binding assays.

The elicitor activity and high affinity binding of the hepta-β-glucoside and related β-glucans are limited to members of the Fabaceae (Ebel, 1998; Fliegmann et al., 2004). Biochemical purification and additional studies indicate the binding proteins from legumes constitute a family of proteins of different sizes (75–150 kDa; Ebel, 1998), with different carbohydrate active domains, one that binds β-glucans and another with glucanase activity capable of releasing elicitor-active fragments from *Phytophthora* cell walls (Fliegmann et al., 2004). What would further strengthen the case for these as physiological receptors for β-glucan-triggered immune responses in soybean is evidence that the binding specificity for diverse oligoglucosides matches their bioactivity as elicitors. To our knowledge corresponding knock-out or knock-down genetic experiments within legumes to corroborate receptor function have not been reported, although the soybean protein expressed in tomato confers binding of the hepta-β-glucoside (Mithofer et al., 2000).

## β-GLUCANS IN IMMUNE SUPPRESSION AND ACTIVATION IN THE SOLANACEAE

β-1→3-glucans also figure prominently as immune modulators in the potato – *P. infestans* interaction, although the story here is complicated by their reported action as both enhancers and suppressors of elicitor activity. However, this differential activity has not been reconciled with the degree of biochemical resolution as was done with *P. sojae* glucans to unambiguously assign enhancer or suppressor activity to the various oligoglucosides within the active fractions. Doke and Tomiyama (1980) using a potato protoplast assay showed that water soluble, anionic and non-anionic β-glucans suppressed the elicitor activity of a crude hyphal wall fraction from *P. infestans*. They suggested a degree of race-specificity in that glucans from compatible races of the pathogen were more active than those of incompatible races in suppressing the HR and ROS induced by the hyphal wall elicitor. The suppressive glucans were partially characterized and shown to have a DP of 17–23 glucose units with β-1→3 and β-1→6 linkages, and were present in the fluids of germinating cystospores (Doke et al., 1979). The purified hepta-β-glucoside from *P. sojae* was neither active as an elicitor nor as a suppressor in potato. A subsequent study showed that water soluble glucans from spore germination fluids of *P. capsici* have similar effect in suppressing elicitor-induced cell death in pepper and tomato cell suspensions (Sanchez et al., 1994). Race specificity attributed to the glucans in the context of HR suppression is difficult to reconcile with the contemporary paradigm of effector-triggered immunity and resistance (R)-gene action (Chisholm et al., 2006).

The model for β-glucans as suppressors is further complicated by their enhancement of EP elicitor activity. β-glucans, although lacking inherent elicitor activity in potato, can strongly enhance the activity of EP. Several lines of evidence suggest the combined action of eliciting (EP) and non-eliciting (β-glucans) components provide a maximal defense response. Initial evidence came from reconstitution experiments whereby highly elicitor-active, solubilized cell wall fractions were hydrolyzed in base-borohydride, leaving polysaccharides intact but hydrolyzing any esterified fatty acids, which were then removed by solvent extraction. This resulted in complete loss of elicitor activity, which was restored by addition of AA and EPA to the base-hydrolyzed wall fractions at their levels initially present (Bostock et al., 1982). Subsequent fractionation, partial purification and analysis showed that the enhancers were indeed β-1→3-glucans (Maniara et al., 1984). Preisig and Kuć (1985) further demonstrated that the glucans provide a 10–100 fold enhancement of the activity of AA concentrations that alone are below the threshold for induction of phytoalexins and related responses. The glucans also revealed elicitor activity of other EPs, particularly Δ5-eicosatrienoic acids. The most active β-glucan fractions had similar DP as the suppressor glucans, and were then found to suppress the HR induced by incompatible races of *P. infestans*, suggesting that the enhancers and suppressors could be the same.

These classic experiments indicate that members of the Solanaceae have an intriguing system for perceiving specific β-glucans and EP to coordinate a strong resistance response. The activity of these glucans in modulating immunity in potato, in particular, suggests a receptor-mediated process subject to attenuation by competing ligands as observed in legumes. For example, the suppressive action of the β-glucans against the HR induced by pathogen inoculum or the crude hyphal wall elicitor may have resulted from similarities in oligosaccharin motifs that compete for a putative MAMP receptor. Algal polysaccharides, such as the storage β-glucans laminarin and carrageenan, activate defense responses in some plants, although sulfated carrageenans appear to be far more active than laminarins as elicitors (Klarzynski et al., 2000; Mercier et al., 2001). However, in potato, laminarin neither elicits nor suppresses, providing a negative control treatment in the studies of the more complex β-1→3-linked glucans (Bostock et al., 1982; Maniara et al., 1984; Preisig and Kuć, 1985). Although considerably less active than the β-glucans, N, N’-diacetyl-D-chitobiose, the hapten for the potato lectin, inhibited the HR induced by incompatible races of *P. infestans* in potato (Nozue et al., 1980) and modestly enhanced the elicitor activity of AA (Maniara et al., 1984). Although other carbohydrates may modulate the plant immune response to some degree, the exceptionally strong biological activity of the oomycete oligosaccharins indicates considerable structural specificity in their action.

## β-GLUCAN RECOGNITION IN ANTIFUNGAL IMMUNITY IN VERTEBRATES

Protection against fungi in vertebrates involves both innate and adaptive immunity (Brown, 2011). Innate antifungal immunity is primarily mediated by diverse pattern-recognition receptors associated with phagocytes, which upon activation ingest and kill or degrade the invading microbe. Carbohydrates associated with the fungal cell wall, in particular, are well positioned to be recognized by these receptors. The adaptive and highly specific immune response to the invader is then engaged following generation of cytokines and chemokines along with the presentation of microbial antigens to lymphocytes.

There are multiple pattern-recognition receptors for β-glucans in phagocytes and the molecular details for some of these interactions have been characterized (Brown and Gordon, 2005). These include the transmembrane dectin-1, a natural killer-cell-receptor-like C-type lectin (calcium dependent) found on macrophages, neutrophils and dendritic cells, which specifically recognizes β-1→3- and β-1→6-linked glucans as well as intact yeast cells (Brown, 2006; Schorey and Lawrence, 2008). Zymosan, a complex cell wall preparation from *Saccharomyces cerevisiea* used to promote inflammation in experimental models, also stimulates dectin-1 and macrophage activation. Of particular interest in relation to the topic of this review is that zymosan induces cytosolic phospholipase A2 in macrophages that releases AA for conversion into pro-inflammatory prostaglandins and leukotrienes (Suram et al., 2006; Olsson and Sundler, 2007). Intriguing here is the apparent cross-kingdom conservation whereby β-glucans operate in concert with AA metabolites and other signals to orchestrate an innate immune response. The extent to which this analogy and underlying mechanisms translate to plant–oomycete interactions remains to be determined. *Arabidopsis* and *Solanum* species have proteins with C-type lectin motifs with some homology to dectin-1. However, they appear to be rare in plants and their functions are unresolved (Singh and Zimmerli, 2013).

## PERSPECTIVES

The “renaissance of elicitors” heralded in the excellent review by Boller and Felix (2009) reflects a raised awareness and renewed interest in some of the classic elicitors. Recasting these as MAMPs has provided a framework that can inform and guide research into their perception and action in plant cells. The extent that different MAMPs collaborate *in vivo* during infection to synergize a strong defense response is unclear, although the cellular machinery seems to be present to do so. The oligomerization of receptors upon MAMP stimulation – the ligand-induced FLS2-BAK1 interaction and coordination with brassinosteroid signaling being a canonical example (Wang, 2012) – should encourage research in other systems for similar examples. It appears that the well-studied receptor-like kinases provide one of several strategies plants use to perceive elicitors to trigger innate immunity (Boller and Felix, 2009; Greeff et al., 2012). A challenge with different MAMPs apparently operating within the same infection interface is that mixed and potentially conflicting messages emanate from phytohormone-regulated response networks, leading to unwanted tradeoffs in the resistance phenotype (Bostock, 2005). How the plant negotiates these trade-offs will be an important consideration.

**FIGURE 5:**
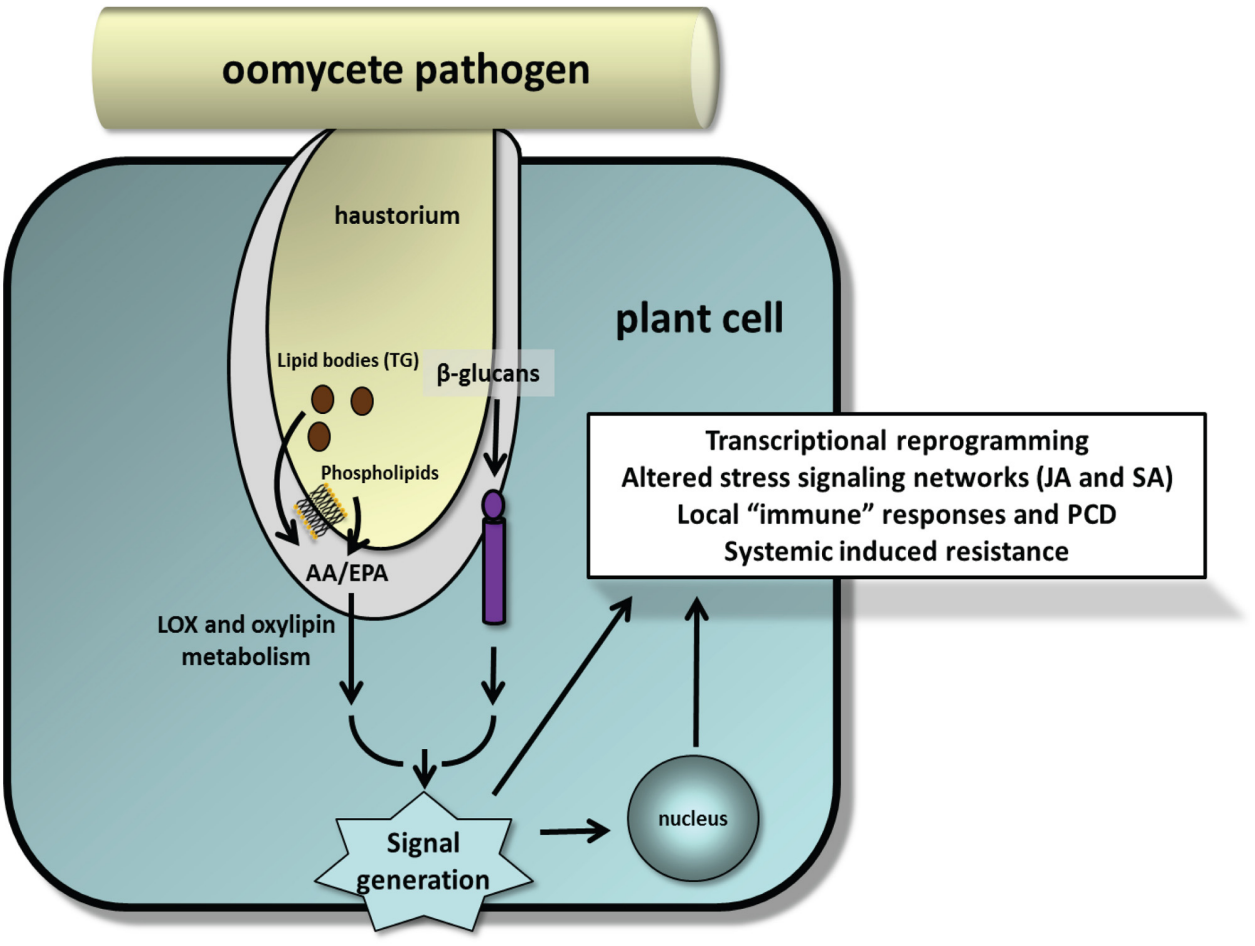
**Model illustrating release of EP from phospholipids and TG-rich lipid bodies and β-glucans during early stages of plant–oomycete interactions as suggested by experimental studies.** Implicit in this model are the fusion of lipid bodies with the haustorial membrane and activation of appropriate lipases and glucanases at the host–parasite interface to release these MAMPs. Minor amounts of EP also can be detected in cell wall fractions. How pathogenic oomycetes suppress these processes for successful infection and colonization is unresolved.

An implicit feature of innate immunity is that MAMPs be presented in their most biologically active form. The β-1→3-glucanase activity of the soybean binding proteins seems to be ideally positioned to release active β-glucan oligomers from invading hyphal walls (Fliegmann et al., 2004), and immunomodulatory glucans from *Phytophthora spp.* can be found in spore germination fluids (Doke et al., 1979; Waldmuller et al., 1992; Sanchez et al., 1994). The overwhelming evidence indicates that EP must be released from esterified forms for them to be perceived to trigger cellular responses (Bostock, 1989; Ricker and Bostock, 1992). A better understanding of how, when and where EP and β-glucans are deployed during the infection and whether they converge to coordinate immune responses will help to fully realize the potential of MAMP-triggered immunity in plant–oomycete interactions (**Figure 5**). With sequenced genomes, technical advances in transcriptomics, proteomics and metabolic profiling, and high-throughput functional assays, now is an opportune time to re-examine these elicitors in crop models.

## Conflict of Interest Statement

The Guest Associate Editor Gitta Coaker declares that, despite being affiliated to the same institution as the authors, the review process was handled objectively and no conflict of interest exists. The authors declare that the research was conducted in the absence of any commercial or financial relationships that could be construed as a potential conflict of interest.

## Footnotes

1 Microbe-associated molecular pattern.

2 In plants, 9-LOXs insert oxygen at the 9-carbon of LA and ALA, which is carbon 1 in the (1Z,4Z)-pentadiene system closest to the carboxyl end of the molecule. The Δ5-carbon of AA is in the carbon 1 position of the (1Z,4Z)-pentadiene system closest to the carboxyl group.

## ABBREVIATIONS

AA: arachidonic acid (20:4 Δ^5,8,11,14^)
ALA: α-Linolenic acid (18:3^Δ^ ^9,12,15^)
AOS: allene oxide synthase
CA: colneleic acid
a divinyl ether from peroxidized LA
CnA: colnelenic acid
a divinyl ether from peroxidized ALA
DES: divinyl ether synthase
DHA: docosahexaenoic acid (22:6 Δ^4,7,10,13,16,19^)
DP: degree of polymerization
DPI: diphenyleneiodonium
EAS: epoxy alcohol synthase
EP: eicosapolyenoic acids (arachidonic acid and/or eicosapentaenoic acid)
EPA: eicosapentaenoic acid (20:5 Δ^5,8,11,14,17^)
ET: ethylene
HMGR: 3-Hydroxy-3-methylglutaryl coenzyme A reductase
HPETE: Hydroperoxyeicosatetraenoic acid
HPL: Hydroperoxide lyase
HPOD: Hydroperoxyoctadienoic acid (from LA)
HPOT: Hydroperoxyoctatrienoic acid (from ALA)
HR: hypersensitive response
JA: jasmonic acid
LA: linoleic acid (18:2^Δ9,12^)
LOX: lipoxygenase
MAMP: microbe associated molecular pattern
PCD: programmed cell death
POX or PXG: peroxygenase
PR: pathogenesis-related (proteins)
PRR: pattern recognition receptor
PTI: pattern triggered immunity
PUFA: polyunsaturated fatty acid
ROS: reactive oxygen species
SA: salicylic acid
SAR: systemic acquired resistance
TG: triglyceride
TMV: tobacco mosaic virus
